# Circular Versus Linear Versus Hand-Sewn Gastrojejunostomy in Roux-en-Y-Gastric Bypass Influence on Weight Loss and Amelioration of Comorbidities: Data Analysis from a Quality Assurance Study of the Surgical Treatment of Obesity in Germany

**DOI:** 10.3389/fsurg.2014.00023

**Published:** 2014-06-23

**Authors:** Christine Elisabeth Stroh, Grigorij Nesterov, Rudolf Weiner, Frank Benedix, Christian Knoll, Matthias Pross, Thomas Manger, Obesity Surgery Working Group, Competence Network Obesity

**Affiliations:** ^1^SRH Municipal Hospital, Gera, Germany; ^2^Institute for Quality Assurance in Surgical Medicine at the University Hospital Magdeburg, Magdeburg, Germany; ^3^Helios Hospital, Bad Saarow, Germany; ^4^Sachsenhausen Hospital, Frankfurt am Main, Germany; ^5^University Hospital Magdeburg, Magdeburg, Germany; ^6^StatConsult, Magdeburg, Germany; ^7^DRK Hospital Berlin-Koepenick, Berlin, Germany

**Keywords:** Roux-en-Y-Gastric Bypass, circular versus linear and hand-sewn anastomosis, amelioration of comorbidities, weight loss

## Abstract

**Background:** Since January 1 2005, the outcomes of bariatric surgeries have been recorded in Germany. All data are registered prospectively in cooperation with the Institute of Quality Assurance in Surgery at Otto-von-Guericke University Magdeburg.

**Methods:** Data are collected in an online data bank. Data collection began in 2005 for the results of Roux-en-Y-Gastric Bypass (RYGB). In addition to primary bariatric operations, data regarding the complications and the amelioration of comorbidities have been analyzed. Participation in the quality assurance study is required for all certified centers in Germany.

**Results:** Roux-en-Y Gastric Bypass is the most popular bariatric operation in Germany. There were 5115 operations performed from 2005 to 2010. A circular anastomosis was performed in 1587 patients, and a linear anastomosis was performed in 2734 patients. In 783 patients, the hand-sewn technique was used. The leakage rate for the linear technique is 1.6%, and the leakage rate is 1.2% for circular anastomosis, and 1.4% for hand-sewn technique.

**Conclusion:** Roux-en-Y-Gastric Bypass is a popular procedure in Germany. The complication rate has decreased since 2005. The amelioration of comorbidities is not influenced by the anastomosis technique. Additional data are necessary to evaluate the long-term effect of linear versus hand-sewn and versus circular-stapled gastrojejunal anastomosis regarding pouch dilatation, small bowel dilatation, and consecutive weight loss.

## Introduction

Obesity is one of the greatest challenges to health in the twenty-first century. According to data from the International Association for the Study of Obesity (IASO), Germany ranks first in the prevalence of obesity for both genders (1). The Federal Office of Statistics revealed that in 2011, 67.1% of men and 53% of women were overweight (2).

Laparoscopic Roux-en-Y-Gastric Bypass (RYGB) was introduced in Germany in the mid-1990s. The long-term durability of RYGB has been shown in different studies. The effects of long-term follow-up over a period of more than 10 years and long-term outcomes after RYGB have recently been published (3). Due to the high BMI of patients in Germany and the high prevalence of comorbidities, the use of RYGB has rapidly increased in Germany in recent years.

In Germany, a stapled anastomosis is more common than the hand sutured anastomosis of the gastrojejunal junction (GJA). In the literature, anastomotic leak rates reach 5.1% with linear staplers and up to 6.6% with circular devices (4).

This study investigated the outcomes with regard on weight loss and amelioration on comorbidities for patients undergoing RYGB with linear or circular staples based on an analysis of data from the German Bariatric Surgery Registry (GBSR). The primary goal of the study was to compare the short-term follow-up rates for the different techniques of gastrojejunal anastomosis in RYGB.

## Method

Since January 1 2005, data from a quality assurance study of the surgical treatment of obesity in Germany have been registered prospectively in an online database at the Institute of Quality Assurance in Surgical Medicine of the Otto-von-Guericke University Magdeburg (5, 6). This study evaluates the outcomes of RYGB as a primary operation for data collected between 2005 and 2010. The data studied include patient’s demographics, surgical parameters, complications, and mortality following RYGB for circular versus linear anastomosis with respect to the incidence of leakage, short-term morbidity, and mortality. The results are compared with other findings in the literature.

The paper analyzed data of GBSR without any selection criteria for the patients to several stapling techniques for gastrojejunal anastomosis or hand-sewn anastomosis. Decision depends on the experience of the different centers in Germany.

To ascertain completeness of data entry, participation in the study is a pre-requisite to an institution’s certification as a center for bariatric surgery, and reported case numbers are verified against hospitals’ clinical and billing documentation. The anonymized data are provided to the institutions and serves a center’s quality control in regards to surgical indication, complication rates, outcome, and follow-up in comparison with other centers. To optimize quality and integrity of registry data (e.g., minimize missing data and ensure adherence to eligibility criteria), statistical plausibility checks are employed and regular random audits are conducted by the study director.

The study period ranged from January 1 2005 to December 31 2010 and 96 centers participated. Patients who underwent a primary bariatric procedure were included in a descriptive statistical analysis performed by an independent statistics consulting firm (StatConsult, Magdeburg, Germany).

The study was conducted according to the Recommendations of the Declaration of Helsinki for Biomedical Research and the Guidelines and Standards of the Institutional Review Boards. All patients provided informed consent prior to entering the study.

## Statistical Analysis of Data

Statistical analysis was performed by StatConsult GmbH using the SAS^®^ 9.2 software program. The descriptive statistical analysis was specified by presentation of absolute and relative frequencies for categorical data and mean, standard deviation, minimum and maximum values for continuous variables. The median was presented for continuous variables with high variation. For further verification of differences between groups in categorical data, the χ^2^ test was used or the Fisher’s exact test was applied for rare events. Continuous variables were compared between two groups using a two sample *t*-test. Multivariable analysis of several influence parameters in parallel for a dependent binary variable was performed using logistic regression. *P*-values were determined for two-sided tests, with a value of *p* < 0.05 indicating a statistically significant difference. All tests were deliberately carried out to the full level of significance.

## Results

There were 5115 RYGBs performed as a primary approach between 2005 and 2010. In relation to gender distribution, there were 3993 female patients and 1122 male patients. In 2734 (53.6%) patients, the GJA was performed with linear staplers, and in 1587 (31.1%) patients, circular staplers were used. Seven hundred eighty-three patients were operated using the hand-sewn technique. For 11 patients, no valid statement could be made.

### Demographic data

The mean age of patients with circular anastomosis was 41.5 years and was not significantly higher than the mean age for patients in the linear group, which was 40.9 years (*p* = 0.084). Patients with a hand-sewn technique had a mean age of 40.2 years.

The mean BMI of the patients in the linear group was 47.6 kg/m^2^ (23.1–73.8 kg/m^2^), and this value was significantly lower than that of the circular-stapled group, which had a mean BMI of 49.8 kg/m^2^ (31.6–85.2 kg/m^2^) (*p* < 0.001). Patients with hand-sewn had a mean BMI of 48.6 kg/m^2^ (27.1–86.0 kg/m^2^) (Table 1).

**Table 1:** **Demographic data**.

	Demographic data			
	Linear	Circular	Stapled (total)	Hand-sewing
Total number of patients (*n*)	2734	1587	4321	783
BMI (kg/m^2^)	47.6 ± 6.7	49.8 ± 7.4	48.4 ± 7.1	48.6 ± 7.8
Age (years)	40.9 ± 10.8	41.5 ± 10.5	41.1 ± 10.7	40.2 ± 10.3
Male (%)	21.8	22.1	21.9	22.2
BMI (kg/m^2^)	48.1 ± 6.6	49.9 ± 7.3	48.8 ± 6.9	49.7 ± 8.0
Age (years)	43.6 ± 11.4	42.6 ± 10.9	43.2 ± 11.2	43.3 ± 10.2
Female (%)	78.2	77.9	78.1	77.8
BMI (kg/m^2^)	47.5 ± 6.8	49.8 ± 7.4	48.3 ± 7.1	48.3 ± 7.7
Age (years)	40.1 ± 10.6	41.2 ± 10.3	40.5 ± 10.5	39.2 ± 10.1

### Comorbidities

Comorbidities were recorded for all patients in the study. The overall incidence of comorbidities was 84.3% (*n* = 4303). Patients with circular anastomosis suffered significantly more comorbidities than patients with linear anastomosis (*p* < 0.001). The occurrence of the different comorbidities is shown in Table 2.

**Table 2:** **Comorbidities for linear versus circular-stapled anastomosis and stapled versus hand-sewing in RYGB**.

	Anastomosis			
	Linear (*N* = 2734)	Circular (*N* = 1587)	Stapled (total) (*N* = 4321)	Hand-sewing (*N* = 783)
Total number of patients with comorbidities (*n*)	2283	1390	3673	630
Overall comorbidities (%)	83.5	87.6	85.0	80.5
*p*-Value	<0.001		0.001	
Male (%)	88.4	89.4	88.8	87.4
*p*-Value	0.619		0.584	
Female (%)	82.1	87.1	83.9	78.5
*p*-Value	<0.001		<0.001	
Hypertension (%)	56.4	59.8	57.7	52.4
*p*-Value	0.031		0.006	
IDDM (%)	8.0	12.2	9.6	8.6
*p*-Value	<0.001		0.367	
NIDDM (%)	20.6	23.6	21.7	18.6
*p*-Value	0.020		0.054	
Sleep apnea (%)	17.3	22.7	19.3	18.6
*p*-Value	<0.001		0.680	
GERD (%)	18.8	17.8	18.4	14.2
*p*-Value	0.446		0.004	
Cardiac disease (%)	5.6	12.9	8.3	5.7
*p*-Value	<0.001		0.015	
Pulmonary disease (%)	13.8	25.6	18.1	19.9
*p*-Value	<0.001		0.231	
Skeletal disease (%)	39.1	54.8	44.9	27.4
*p*-Value	<0.001		<0.001	

### Follow-up data

Follow-up data are available for 2246 patients. For patients with circular anastomosis, the follow-up rate was 47.8% (*n* = 759), for linear staples 39.4% (*n* = 1078). The follow-up rate for patients with hand-sewn procedure was 52.2% (*n* = 409).

### BMI reduction

The mean BMI reduction for patients with linear-stapled GE was 14.74 kg/m^2^. The mean BMI reduction for patients with circular anastomosis was 15.10 kg/m^2^. A comparison of both stapler groups showed no significant difference (*p* = 0.193). Patients with hand-sewn GJA had a mean BMI reduction of 13.69 kg/m^2^, which is significant lower than for patients with linear and circular-stapled GJA (*p* < 0.05).

### Amelioration of comorbidities

There were 212 patients with available follow-up data prior to surgery regarding insulin-dependent DM II. Preoperatively, 1269 patients suffered from hypertension and 449 patients suffered from sleep apnea. The overall rate of remission or reduction of hypertension was 80.1%. The remission/reduction rate for all patients with sleep apnea is 80.4%.

## Discussion

Since January 1, 2005, primary and revisional bariatric procedures have been recorded within the framework of a quality assurance study of the surgical treatment of obesity by the Institute for Quality Assurance in Surgical Medicine at the Otto-von-Guericke University Magdeburg with the aim of improving the quality of care (5, 6).

Roux-en-Y-Gastric Bypass is one of the most frequently performed bariatric operations worldwide. For RYGB complications, a leak at the GJA is the most critical aspect to judge the integrity of the anastomosis. The presence of bleeding, marginal ulceration, or anastomotic strictures are of secondary importance.

## Loss of BMI

During a median follow-up of 13 months (range 19–2090 days), the mean BMI loss for patients with linear GJA was 14.74 kg/m^2^. For patients with circular-stapled GJA, the mean BMI loss was 15.10 kg/m^2^. The difference is not statistically significant. These data are similar to the published data form a controlled randomized study by an Austrian group (7). Similar to the literature, both groups reach the BMI nadir 2 years after surgery. Meta-analysis data did not indicate a significant difference between the two techniques concerning the excess weight loss (8). Patients with hand-sewn GJA had a mean BMI reduction of 13.69 kg/m^2^, which is significantly lower than for patients with linear or circular-stapled GJA (*p* < 0.05).

## Amelioration of Comorbidities

### Hypertension

Following RYGB, 80.08% of all patients (83.95% in the linear group, 75.11% in the circular group, and 78.95% in hand-sewn) had a decrease in hypertension. The reduction rate was 33.23% (37.81% linear versus 30.57% circular, and 24.88% for hand-sewn), and the amelioration rate was 46.84% (46.14% linear versus 44.54% circular, and 54.07% for hand-sewn procedure). The literature reports an amelioration rate of hypertension after RYGB between 22 and 100% (9–11). The problems with these studies and the GBSR data are the absence of investigations into the duration of hypertension or the lack of correlations with age, gender, and BMI.

### Insulin-dependent diabetes type II

The remission rate of IDDM was 56.23% (59.76% linear versus 54.00% circular, and 55.10% for hand-sewn), and a reduction of insulin was found in furthermore 27.27% of patients (30.49% linear versus 29.00% circular, 18.37% for hand-sewn).

Patients with linear anastomosis had a higher remission rate than patients with circular anastomosis. Further investigations evaluating this effect are necessary. Investigations into the duration of remission of insulin-dependent diabetes type II are also necessary to evaluate the long-term metabolic effect of RYGB. These investigations should include analysis of diabetes duration, duration of insulin treatment, insulin dose, age, gender, and BMI.

### Non-insulin-dependent diabetes mellitus type II

Data from GBSR have shown the decrease of non-insulin-dependent diabetes type II was 85.00% for all patients. The reduction occurred in 92.13% of patients with linear-stapled GJA, 78.11% reduction occurred in patients with circular-stapled GJA, and 76.62% for hand-sewing procedure. The reduction rate was 24.20% overall (26.77% linear versus 18.34% circular GJA, and 28.57% for hand-sewn), and the additional complete remission rate was 60.80%. The complete remission was 65.35% in patients with linear-stapled GJA and 59.76% in patients with circular anastomosis and 48.05% for hand-sewn. Patients with linear anastomosis have shown a better reduction and remission rate than patients with circular GJA. The bias of our data is that patients with circular GJA suffered preoperatively from more comorbidities than patients with circular anastomosis. Another problem is that we did not assess the preoperative duration of diabetes for evaluation of remission rate. Further studies and investigations of GBSR should analyze this limitation (6).

Data in the literature report a reduction rate of 69–100% after RYGB with a complete remission rate of approximately 80% (9, 12).

The GBSR results include several limitations because patients in Germany have a higher BMI, older age, and suffer from more comorbidities than patients in most studies published worldwide. These preoperative data influence the results and outcome of the German patients (6).

### Sleep apnea

A decrease of sleep apnea occurred in 80.38% of all patients (84.69% linear versus 79.58% circular, and 70.27% for patients with hand-sewing procedure) with a complete remission of OSA Syndrome in 46.41% of all patients (48.33% linear versus 43.98% circular, 47.30% for hand-sewn).

The literature data show an amelioration rate of 15% in the SOS-Study and 86.6–100% in the meta-analysis performed by Buchwald (9, 10).

## Conclusion

The data from the GBSR on the surgical treatment of RYGB have not shown any significant differences in the leakage and stricture rates of gastrojejunal anastomosis when comparing linear versus circular techniques according to data published from the meta-analysis (7, 9).

Using linear or circular stapler devices does not significantly influence weight loss postoperatively or alter the amelioration of sleep apnea and hypertension comorbidities. For patients with diabetes mellitus type II, we found a better amelioration rate in linear anastomosis, which is in contrast to meta-analysis. However, the bias of our study is the higher prevalence of comorbidities and the higher BMI in patients with circular-stapled anastomosis and the missing randomization of our patients (8).

Further examinations are necessary to investigate the long-term effect of complete remission of diabetes mellitus type II and hypertension.

The bias and limitations of the GBSR include the evaluated center effect, the high BMI, the incidence of comorbidities of patients in Germany, and the lack of data on duration and treatment of comorbidities and their influence on amelioration. Further studies are necessary to evaluate the complication rates of different stapler devices and assess the impact on amelioration of comorbidities and their long-term effects.

## Conflict of Interest Statement

The German Nationwide Survey on Bariatric Surgery is supported by the Ministry of Research and Education Germany (BMBF) grant number 01GI1124. The responsible investigator is Christine Elisabeth Stroh. Christine Elisabeth Stroh, Grigorij Nesterov, Rudolf Weiner, Frank Benedix, Christian Knoll, Matthias Pross, and Thomas Manger confirm that there are no links to firms whose products are mentioned in the article or to firms marketing a competing product. The topic is presented in an independent manner, and the information outlined is product neutral. The following firms support the GBSR without any relation to their products: Johnson & Johnson MEDICAL GmbH, Ethicon Endo-Surgery Deutschland, Norderstedt. Covidien Deutschland GmbH Neustadt/Donau.
